# Strong HLA-DR expression in microsatellite stable carcinomas of the large bowel is associated with good prognosis

**DOI:** 10.1038/sj.bjc.6600507

**Published:** 2002-09-23

**Authors:** T Løvig, S N Andersen, L Thorstensen, C B Diep, G I Meling, R A Lothe, T O Rognum

**Affiliations:** Institute of Forensic Medicine, The National Hospital, University of Oslo, 0027 Oslo, Norway; Department of Genetics, Institute for Cancer Research, The Norwegian Radium Hospital, 0310 Oslo, Norway

**Keywords:** colorectal neoplasms, HLA-DR, immunohistochemistry, microsatellite instability, survival

## Abstract

Progression of colorectal cancer may follow either of two main genetic routes: the chromosome- or microsatellite-instability pathways. Association between the patients' prognosis and microsatellite instability has been questioned. Improved survival has previously been found in patients with expression of HLA-DR antigens on their tumour cells. In this study, the expression of HLA-DR antigen was investigated by immunohistochemistry in 357 large bowel carcinomas stratified by microsatellite instability status. Sixteen per cent of the tumours showed strong HLA-DR expression and 35% had weak DR expression. We confirmed that patients with strong positive HLA-DR staining had improved survival (*P*<0.001) compared to patients with no HLA-DR expression. Strong epithelial HLA-DR staining was significantly associated with high level of microsatellite instability (*P*<0.001). In the subgroup of tumours with characteristics typical of the chromosomal instability phenotype, i.e. in microsatellite-stable tumours, the patients positive for the HLA-DR determinants showed better survival than those without HLA-DR expression. The protective effect of HLA-DR expression on survival was confirmed by multivariate analysis, both in the whole patient group and in the microsatellite-stable/microsatellite instability-low group. This might be explained by enhanced T-cell mediated anti-tumour immune responses against tumour cells in the HLA-DR positive tumours. The finding of better patient survival in the subgroup of strong HLA-DR positive microsatellite-stable tumours may have clinical implications for these patients.

*British Journal of Cancer* (2002) **87**, 756–762. doi:10.1038/sj.bjc.6600507 www.bjcancer.com

© 2002 Cancer Research UK

## 

Primary colorectal tumours may be divided into two main groups related to the genetic profile of the tumour (Kinzler and Vogelstein, 1996). Tumours with chromosome instability (CIN) reveal high rates of chromosome losses and gains, whereas tumours with microsatellite instability (MSI) show genome-wide changes in repetitive sequences due to defects in the DNA mismatch repair system. Most tumours in hereditary non-polyposis colorectal cancer (HNPCC), and about 15% of sporadic colorectal carcinomas show MSI (Aaltonen *et al*, 1993; Lothe *et al*, 1993). MSI leads to the accumulation of deletion and insertion of nucleotides at simple repeat sequences. The clinical and pathological features of colorectal cancers showing high degree of MSI (MSI-H) are proximal location, poor differentiation, mucinous phenotype, high density of lymphocyte infiltration, diploid DNA content, reduced invasiveness, female preponderance and multiplicity (Lothe *et al*, 1993; Kim *et al*, 1994; Ruschoff *et al*, 1995; Messerini *et al*, 1997; Jass *et al*, 1998; Thibodeau *et al*, 1998). High density of tumour infiltrating lymphocytes (TIL) are found in MSI-H colorectal cancers, whereas it is only infrequently observed in MSI-L and MSS tumours (Jass *et al*, 1998; Smyrk *et al*, 2001). Recently, two studies have identified significantly more cytotoxic lymphocytes (CD3, CD8 and TIA-1) and a higher rate of cells undergoing apoptosis in MSI-H colorectal carcinomas than in MSI-L and MSS (Dolcetti *et al*, 1999; Michael-Robinson *et al*, 2001). Furthermore, patients with MSI-H tumours and a high content of activated cytotoxic lymphocytes showed improved overall survival (Guidoboni *et al*, 2001). Some studies have reported improved survival among colorectal cancer patients with microsatellite unstable tumours (Lothe *et al*, 1993; Thibodeau *et al*, 1993; Bubb *et al*, 1996; Lukish *et al*, 1998; Halling *et al*, 1999; Johannsdottir *et al*, 1999; Massa *et al*, 1999; Elsaleh *et al*, 2000; Gryfe *et al*, 2000; Hemminki *et al*, 2000; Wright *et al*, 2000; Ward *et al*, 2001; Watanabe *et al*, 2001), while others have not (Messerini *et al*, 1997; Senba *et al*, 1998; Feeley *et al*, 1999; Salahshor *et al*, 1999; Curran *et al*, 2000).

The major histocompatibility complex (MHC) in humans plays an important role in various kinds of immune reactions, including antigen presentation, cytotoxic response, and immune recognition. The MHC encodes for three classes of cell antigens: class I (HLA-A, -B, -C), class II (HLA-DR, -DQ, -DP) and class III (Klein and Sato, 2000). HLA-DR antigen expression is usually not present in the epithelium of the large bowel, but may be turned on in inflammatory disorders, such as ulcerative colitis (Rognum *et al*, 1987), Crohn's disease and in colorectal tumours (Rognum *et al*, 1983; Gutierrez *et al*, 1987; Norheim Andersen *et al*, 1993; Chang and Sohn, 1995; Lazaris *et al*, 1995; Morita *et al*, 1995; Kunihiro *et al*, 1998). HLA-DR antigen expression on tumour cells is a marker of good prognosis in large bowel carcinomas (Gutierrez *et al*, 1987; Norheim Andersen *et al*, 1993; Lazaris *et al*, 1995; Morita *et al*, 1995; Kunihiro *et al*, 1998).

In a large series of primary colorectal carcinomas, stratified as MSI-H and MSS/MSI-L, we have analysed the epithelial expression of HLA-DR and addressed the association with clinical and pathological variables.

## MATERIALS AND METHODS

### Patients

The study was based on two series of tumours collected from hospitals in the Oslo and Akershus region. The first series of 100 large bowel carcinomas from 100 patients were operated on between 1978 and 1982, and have previously been studied according to HLA-DR expression (Norheim Andersen *et al*, 1993). The second consecutive series of tumours from 257 patients with primary colorectal cancer were collected during the period 1987–1989. The clinicopathological characteristics of the patients are given in Table 1

**Table 1:**
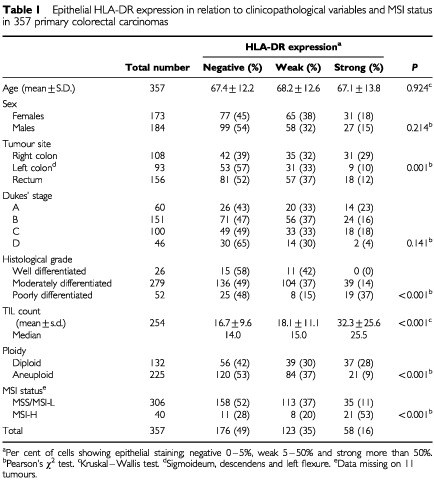
Epithelial HLA-DR expression in relation to clinicopathological variables and MSI status in 357 primary colorectal carcinomas

. The median age at diagnosis was 67 years for males (range 26–94) and 69 years for females (range 24–92). Survival was recorded from the date of resection of the tumour until death or until the censor date (1 July 1995 for the first series of 100 patients and 1 July 1999 for the second series of 257 patients). Patients that died within 30 days after operation were censored and cancer-specific deaths have been recorded from hospital and post-mortem reports. All of the patients underwent surgery alone as the curative treatment, except for a few patients with rectal cancers who also received postoperative radiation therapy. Two patients in the second series of tumours fulfil the Amsterdam criteria for HNPCC, one case being MSS and the other MSI-H. For the first series of tumours information about Amsterdam criteria is not available.

### HLA-DR immunohistochemistry

Tissue slices from each of the 257 tumours in the second series were fixed in cold 96% ethanol and processed for paraffin embedding as previously described (Brandtzaeg, 1974). Sections cut at 6 μm were dewaxed and subjected to immunofluorescence staining and one section was stained by a routine method (HAS) containing haematoxylin, azofloxin and saffron. A murine monoclonal antibody to a non-polymorphic human HLA-DR antigen was applied in an indirect three-step immunofluorescence method as described previously (Norheim Andersen *et al*, 1993). The method included affinity purified biotinylated horse anti mouse IgG and fluorecein isothiocyanate (FITC)-labelled avidin.

The epithelial staining for HLA-DR antigens in the carcinomas was evaluated by a Leitz Aristoplan fluorescence microscope (Leica). The tumours were divided into four groups according to the percentage of positive tumour epithelium, 0–5, 5–20, 20–50 and >50%, respectively. In the further analysis, the samples with 0–5% positive cells were considered negative, whereas samples with 5–50% positive cells were classified as weak HLA-DR positive, and those >50% as strong HLA-DR positive. Three sections from 97 tumours and two sections from 224 tumours were investigated, while only one section was investigated in the remaining 36 cases. The same investigator (SNA) was responsible for the fluorescence scoring throughout both series of tumours studied. The HLA-DR staining was scored blindly in view of MSI status of the tumour. Since the scoring procedure showed good inter- and intra-observer reproducibility in the first study of 100 carcinomas (Norheim Andersen *et al*, 1993), such tests were not included in the evaluation of the 257 tumours.

### Histopathological analysis of the tumours

For all tumours, histopathological type and degree of differentiation (WHO classification (Jass and Sobin, 1989)), as well as clinicopathological stage, have been published previously (Meling *et al*, 1991; Rognum, 1991). In the second series of tumours, tumour infiltrating lymphocytes (TIL) were identified by light microscopy on HAS-stained sections as cells with the morphology of lymphocytes, seen within the tumour epithelium. TIL's were counted in 10 high power fields from each tumour, since a stable mean of number of TIL per high power field was achieved in the tumours after counting 10 fields. Enumeration of TIL's was performed by the same experienced investigator (SNA) throughout the study.

### MSI status

The MSI status of the 100 tumours in the first series were analysed by the two mononucleotide repeats, BAT-25 and BAT-26, which are robust markers for MSI-H detection (Loukola *et al*, 2001; Zhou *et al*, 1998). Tumours with mutation in both markers were defined MSI-H (10 cases) and the rest were put in the MSS/MSI-L group.

MSI status in the second series was available in 246 of the 257 tumours (Lothe *et al*, 1993; Thorstensen *et al*, 2001; Diep *et al*, 2002, unpublished). The tumours have been analysed with BAT-25 and BAT-26, in addition to a total of 19 dinucleotide repeats. DNA from each tumour, except from five tumours, was informative in at least five (median 18, range 5–21) of the loci analysed (Thorstensen *et al*, 2001; Diep *et al*, 2002, unpublished). The 30 tumours with instability in ⩾30% of the microsatellite loci analysed, or with mutation in both BAT-25 and BAT-26 were defined as MSI-high (MSI-H). The 32 tumours with instability in at least one locus and less than 30% of the loci was MSI-low (MSI-L), whereas the 184 tumours without microsatellite instability was classified as microsatellite stable (MSS), according to the consensus criteria for classification of MSI (Boland *et al*, 1998).

### Statistics

Statistical analyses were conducted using the SPSS software v10.0. Comparisons between categorical variables were performed using Pearson's χ^2^ analysis. Continuous variables (age and TIL count) were analysed using the non-parametric Kruskal–Wallis test. Survival curves were estimated by the Kaplan-Meier method, and differences were assessed using the log rank test. To estimate the effect of different factors on survival, a multivariate analysis was performed using the Cox proportional-hazards method. The following variables were included in the analysis: age, sex, Dukes' stage, tumour site, histological grade, DNA ploidi pattern, HLA-DR staining and MSI status. A probability value of less than 0.05 was considered statistically significant.

## RESULTS

### HLA-DR expression in relation to MSI status and clinicopathological variables

**Figure 1:**
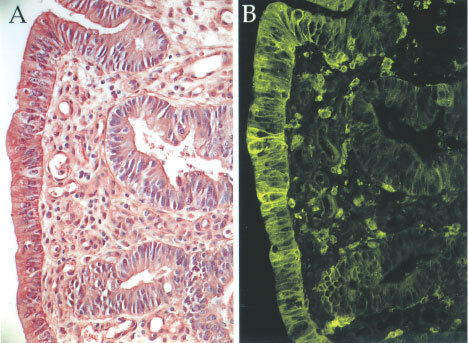
Moderately differentiated large bowel carcinoma with strong HLA-DR expression. (**A**) HAS staining and (**B)** adjacent section stained green for HLA-DR determinants.

Epithelial HLA-DR expression in varying degrees was found in 181 of the 357 (51%) tumours investigated (Figure 1

). Fifty-eight (16%) showed strong HLA-DR expression, whereas 123 (35%) had weak DR expression. HLA-DR expression in relation to MSI status and clinicopathological variables is shown in Table 1. Strong HLA-DR expression occurred in 21 of 40 (53%) of MSI-H tumours, whereas the same was found in only 35 of 306 (11%) MSS/MSI-L tumours (*P*<0.001). Furthermore, tumours with DR expression were related to other variables typical for MSI-H tumours. That is, tumours with strong HLA-DR expression were significantly more often poorly differentiated (*P*<0.001), had more often a diploid DNA pattern (*P*<0.001) and were more frequent located in the right colon than in the left colon or rectum (*P*=0.001) (Table 1). Left-sided and rectal tumours had an equal distribution of strong DR expression. Strong DR positive tumours had significantly higher number of TIL than tumours with weak or no DR-staining (*P*<0.001). There was no association with HLA-DR expression according to Dukes' stages (*P*=0.141).

### HLA-DR expression and survival analysis

**Figure 2:**
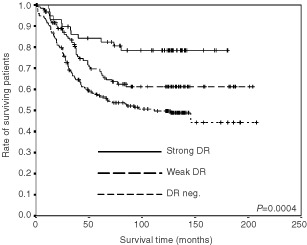
Survival analyses (cancer-related Kaplan–Meier plots) of patients with different degrees of HLA-DR expression. *n*=58 with strong DR expression, *n*=123 with weak DR expression and *n*=176 DR negative.

**Figure 3:**
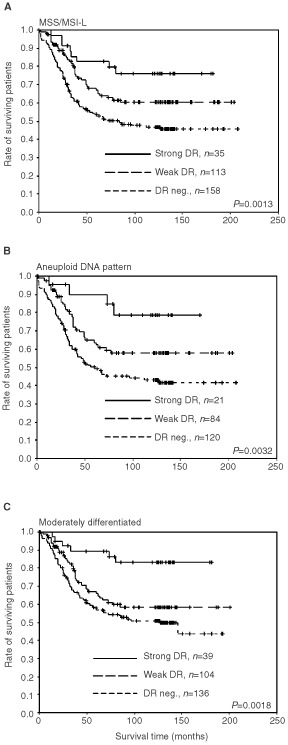
Survival analysis (cancer-related Kaplan–Meier plots) of patients in relation to HLA-DR expression and different variables. (**A**) Patients with MSS/MSI-L tumours, (**B**) patients with aneuploid tumours and (**C**) patients with moderately differentiated tumours. When considering strong DR expression compared to no expression, the *P*-value for MSS/MSI-L tumours was *P*=0.0024, *P*=0.0050 for aneuploid tumours, and *P*=0.0006 for moderately differentiated tumours.

During the follow-up period of 10–13 years there were 138 cancer specific deaths in the group of 357 patients included in the study. Dukes' stage is significantly associated with patient survival (*P*<0.001, log rank test for trend). There is a better patient survival in MSI-H tumours compared to MSS/MSI-L tumours, as shown by both univariate Kaplan–Meier method (*P*=0.004, log rank test for trend) and by multivatiate analysis (HR 0.44, 95% CI: 0.20–0.96, data not shown). Analysis of survival according to HLA-DR in the whole group of patients showed significant differences between the three patterns of tumour HLA-DR expression (*P*<0.001) (Figure 2

). Survival was significantly better in patients with tumours showing HLA-DR expression compared to those with no DR expression, both in the weak- and strong DR expression group (*P*=0.025 and *P*<0.001, respectively). Stratified survival analysis according to sex showed that both males and females with DR-positive tumours had better survival compared to those without (*P*<0.001, data not shown). According to tumour site, stratified analysis showed that patients with HLA-DR positive tumours located in colon had better survival, whereas for patients with rectal tumours DR expression had no influence on patient survival (*P*<0.001 and *P*=0.421, respectively; data not shown). Stratified survival analysis according to tumour MSI status, histological grade and ploidi showed that expression of HLA-DR in tumours was associated with better survival in the subgroups of patients with characteristics typical for the chromosome instability (CIN) phenotype, i.e. MSS/MSI-L tumours. Patients with tumours expressing HLA-DR showed significantly better survival when the tumours were MSS/MSI-L (*P*=0.001) (Figure 3

). The same was true for patients with tumours that had aneuploid DNA content (*P*=0.003) or were moderately differentiated (*P*=0.002) (Figure 3). The good prognosis in the patients with strong HLA-DR positive MSS tumours is not due to higher TIL count in these tumours, as they had a median TIL count of 16.

Survival analysis stratified according to Dukes' stage showed an overall significant difference in survival (*P*=0.003, data not shown).

Multivariate analysis using Cox proportional hazard regression analysis confirmed that both weak and strong HLA-DR expression had an overall protective effect on survival (Table 2

**Table 2:**
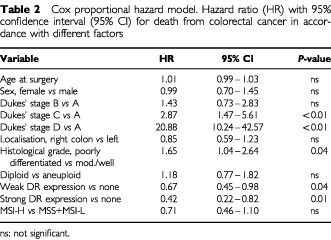
Cox proportional hazard model. Hazard ratio (HR) with 95% confidence interval (95% CI) for death from colorectal cancer in accordance with different factors

). When analysing only the MSS/MSI-L group, the protective effect of DR-expression is still significant (strong HLA-DR expression: HR 0.38, 95% CI: 0.175–0.812 and weak HLA-DR expression: HR 0.68, 95% CI: 0.458–0.998; data not shown).

## DISCUSSION

In the present study we found that strong epithelial HLA-DR expression was highly associated with the MSI-H phenotype. Thus, 53% of the MSI-H tumours were strong HLA-DR positive, *vs* 11% in the MSS/MSI-L group. Because of the strong association between MSI-H tumours and HLA-DR expression, significant correlation was also demonstrated between DR expression and tumours with right-sided location, poor differentiation and diploid DNA pattern, which are the characteristics typical for colorectal tumours following the MSI pathway (Lothe *et al*, 1993; Kim *et al*, 1994; Ruschoff *et al*, 1995; Messerini *et al*, 1997; Jass *et al*, 1998; Thibodeau *et al*, 1998). Strong HLA-DR positive tumours also had a higher TIL count than the other two groups. The presence of an inflammatory reaction in the form of TIL is most frequently found in MSI-H carcinomas (Jass *et al*, 1998; Smyrk *et al*, 2001). No association was found between Dukes' stage and HLA-DR expression, which is consistent with previous studies (Lazaris *et al*, 1995; Kunihiro *et al*, 1998).

The MSI-H tumour phenotype is caused by defects in the DNA mismatch repair machinery, and stretches of repetitive sequences are prone to mutations. When such repeats are present in the coding region, mutations may cause disturbances in normal cellular control. Indeed, frameshift mutations in MSI-H tumours have been reported for several downstream target genes (Ionov *et al*, 1993; Thorstensen *et al*, 2001). The resulting shift in the reading frame of the genes gives rise to syntheses of truncated proteins that have lost all or part of their function. These antigenic peptides could elicit specific anti-tumour immune responses potentially effective in limiting tumorigenesis. This hypothesis is supported by the fact that there is a significant increase of the pronounced inflammatory reaction in the MSI-H tumours. Recently, two studies have identified the majority of TIL in MSI-H tumours as CD8+ T-cells, the number being higher than in MSS tumours (Dolcetti *et al*, 1999; Michael-Robinson *et al*, 2001). The number and distribution of infiltrating CD4+ lymphocytes were similar in MSI and MSS tumours (Dolcetti *et al*, 1999). However, our finding of HLA-DR expression in 53% of the MSI-H tumours, indicate that this might be an important part of the ongoing immune activation supposed to be present in MSI-H tumours. The abnormal peptides produced by MSI-H tumours may be presented to CD4+ T-cells by the HLA-DR molecules expressed on the cell surface. In fact, one recent study has identified a patient with an HLA-DR restricted CD4+ T-cell response against a TGFβRII frameshift peptide (Sæterdal *et al*, 2001). The tumour had heterogeneous HLA-DR expression with prominent CD4+ T-cell infiltration in areas positive for HLA-DR antigen, whereas relatively few cytotoxic CD8+ T-cells were present. This indicates local cytokine production and may be a direct manifestation of immune surveillance.

Patients with HLA-DR positive carcinomas showed significantly better overall survival than in patients with DR negative carcinomas, which is consistent with several other studies (Gutierrez *et al*, 1987; Norheim Andersen *et al*, 1993; Lazaris *et al*, 1995; Morita *et al*, 1995; Kunihiro *et al*, 1998). Quite surprisingly, stratified analyses of survival with respect to MSI status, differentiation, ploidi and tumour localisation disclosed that in MSS/MSI-L tumours, in tumours with aneuploid DNA pattern and in moderately differentiated tumours, strong HLA-DR expression turned out to be a marker for favourable prognosis (Figure 3A,B,C). However, HLA-DR expression in the poorly differentiated, diploid, MSI-H tumours had no influence on survival. The overall protective effect of the HLA-DR expression on survival was confirmed by the multivariate analysis. Also, when analysing only patients with MSS/MSI-L tumours by multivariate analysis, HLA-DR expression had a positive impact on patient survival.

Tumours with the CIN phenotype (usually MSS) represent the majority of sporadic colorectal cancers and are characterised by high rates of chromosome losses and gains. The HLA complex is located on chromosome 6p21, a region infrequently gained in colorectal carcinomas (Bardi *et al*, 1993; de Angelis *et al*, 1999; Rooney *et al*, 1999). The increased level of HLA- DR expression detected in 11% of the MSS tumours is not likely to be explained by a gain of chromosome 6p.

Most studies report that there is an association between MSI-H tumours and improved disease outcome (Lothe *et al*, 1993; Thibodeau *et al*, 1993; Bubb *et al*, 1996; Lukish *et al*, 1998; Halling *et al*, 1999; Johannsdottir *et al*, 1999; Massa *et al*, 1999; Elsaleh *et al*, 2000; Gryfe *et al*, 2000; Hemminki *et al*, 2000; Wright *et al*, 2000; Ward *et al*, 2001; Watanabe *et al*, 2001). Furthermore, several studies have found that the degree of lymphocyte infiltration correlates with a better patient survival (Jass, 1986; di Giorgio *et al*, 1992; Ropponen *et al*, 1997; Naito *et al*, 1998). A recent study found that patients with MSI-H tumours with a high content of activated intra-tumour cytotoxic lymphocytes had better prognosis (Guidoboni *et al*, 2001). This finding supports the presence of an anti-tumour immune response in the MSI-H tumours, which probably explains the better prognosis in these patients. Patients with MSI-H tumours in the present study showed better survival than patients with MSS/MSI-L tumours. However, HLA-DR expression did not turn out to be of prognostic significance in the MSI-H tumours, although about half of the MSI-H tumours showed HLA-DR expression.

In conclusion, HLA-DR expression is most frequently found in carcinomas with MSI phenotype. However, it is also highly expressed in a small subgroup among tumours with the CIN phenotype. These patients have prolonged survival, indicating that HLA-DR expression may be an important part of the anti-tumour immune responses in colorectal carcinomas.
